# F49. EFFECTIVENESS OF THE META-COGNITIVE TRAINING IN PEOPLE WITH FIRST-EPISODE PSYCHOSIS: DOES GENDER MATTER?

**DOI:** 10.1093/schbul/sby017.580

**Published:** 2018-04-01

**Authors:** Susana Ochoa, Miriam Salas, Raquel Lopez-Carrilero, Esther Pousa, Ana Barajas, Eva Grasa, Maria Luissa Barrigón, Esther Lorente, Jordi Cid, Fermin González, Isabel Ruiz, Irene Birulés

**Affiliations:** 1 Parc Sanitari Sant Joan de Deu; 2 Parc de Salut Mar; 3 Les Corts; 4 Hospital Sant Pau; 5 Servicio de Psiquiatria y Psicologia Clinica; 6 Hospital Clínico de Valencia; 7 Institut de Psiquiatria Girona; 8 University of Jaen; 9 University of Málaga

## Abstract

**Background:**

The Meta-Cognitive Training (MCT) is a psychological intervention that combines psychoeducational components and cognitive behavioural therapies with a meta-cognitive approach. The MCT (Moritz and Woodward, 2007) focuses on different cognitive biases, theory of mind and attribution bias, as well as on the predictive value of depressed mood and low self-esteem on paranoid ideation. The MCT has result effective for people with schizophrenia in order to improve symptoms, cognitive insight, jumping to conclusions, memory and quality of life. However, less information is regarding the effectiveness of MCT in first episode psychosis. On the other hand, gender has an important role in psychosis, nevertheless the effect of gender in the effectiveness of MCT has not been proved.

The aim of the study is to assess the effectiveness of MCT regarding symptoms and cognitive insight in people with first episode psychosis, considering the effect of gender.

**Methods:**

A multicenter, randomized, controlled clinical trial was performed. A total of 122 patients were randomized to an MCT or a psycho-educational intervention. The sample was composed of people with a recent onset of psychosis, recruited from 9 public centers in Spain. The treatment consisted of 8 weekly sessions for both groups. Patients were assessed at three time-points: baseline, post-treatment, and at six months of follow-up. The evaluator was blinded to the condition of the patient. Symptoms were assessed with the PANSS and cognitive insight with the BCIS. A regression model for repeated measures was performed with the SPSS by gender.

**Results:**

The sample was composed by 85 men and 37 women, although 53 men and 21 women completed the treatment and the follow-up.

Both psychoeducational and MCT group improved in positive symptoms at post-treatment and follow-up (p<0.05-0.001) with higher effect sizes in the MCT group (0.53 versus 0.38). Regarding negative symptoms the MCT group improved in the follow-up (p<0.001) and general symptoms MCT improved in the post-treatment and follow-up (p<0.001). Cognitive insight was higher in people who attended the MCT, in self-certainty in the post-treatment (p<0.05), self-reflectiveness in the follow-up (p<0.05) and the composite index in both assessments (p<0.05).

Considering the results by gender, men of both groups improve more in positive, negative and disorganized symptoms of the PANSS (p<0.001- 0.046) while women improve in positive symptoms. A tendency of interaction between group and affective symptoms was found only in women (p=0.062), improving more women of the MCT group. Regarding cognitive insight, women of the MCT group improve more in self certainty and total BCIS compared with the psychoeducational group (p<0.001–0.022).

**Discussion:**

MCT could be an effective psychological intervention for people with a recent-onset of psychosis for the improvement of cognitive insight and psychotic symptoms. It seems that women could benefit more from the MCT intervention than men in reduction of affective symptoms and in the improvement of cognitive insight.

